# Investigating the effectiveness of preventive measures to reduce mortalities of drownings from 2017 to 2021 in Alborz province

**Published:** 2023-12

**Authors:** Ahmad Mahdavi, Razieh Kormi, Kivan Haj Mahmoudi, Neda Mohammadi Nahr Khalji, Abolfazl Arghandepour, Abuzar Saeedi

**Affiliations:** ^*a* Emergency Medical Service (EMS), Karaj, Alborz, Iran. ^; ^*b* EMS 115, Karaj, Alborz, Iran. ^

## Abstract

**Background:**

Drowning is one of the unnatural but preventable causes of the death of about 1200 people in Iran every year. According to the official statistics published by the Forensic Medicine Organization of Alborz province from 2019 to 2020, Alborz is one of the provinces with the highest number of drowning deaths. Therefore, it is necessary to take preventive measures to prevent and reduce this number. Preventive measures have been taken during the years 2019-2021 in cooperation with the provincial crisis management council and other institutions. This study aimed to evaluate the effectiveness of these measures with statistical analysis.

**Methods:**

This is a retrospective study of the statistics of emergency missions based on the Asayar system and based on the statistics provided by the Forensic Medicine Organization during the years 2018 (before the intervention), 2019, and 2021 (after the intervention). The results were analyzed using SPSS software (version 16).

**Results:**

According to the data of the Asayar system, during 2018, 54 missions carried out on the subject of drowning were recorded excluding the duplicate cases and canceled missions. This statistic reached 59 in 2019 and 67 in 2020. According to the statistics published by the National Forensic Medicine Organization, the number of deaths due to drowning in Alborz province was 34 in 2018, 44 in 2019, 52 in 2020, and 64 in 2021.

**Conclusions:**

Despite the intervention measures adopted in 2019 and the implementing ideas such as identifying drowning-prone areas, setting up an emergency room on Chalus Road, and the extension of these measures during the years 2020 and 2021, the number of emergency missions and deaths has increased up to 68.75% in comparison with the aggregate statistics of 2016-2018 and 2019-2021. It seems that preventive measures need to be seriously improved to increase their effectiveness.

**Keywords:**

Drowning, Alborz, EMS, Preventive measures

